# Evaluation of an Occupational Exercise Training Program for Firefighters: Mixed Methods Pilot Study

**DOI:** 10.2196/17835

**Published:** 2020-09-21

**Authors:** Miriam Leary, James Thomas, Ryan Hayes, Lori Sherlock

**Affiliations:** 1 Division of Exercise Physiology Department of Human Performance and Applied Exercise Science West Virginia University Morgantown, WV United States

**Keywords:** firefighters, exercise, mixed methods, qualitative, clinical, performance

## Abstract

**Background:**

Occupational exercise training programs can improve overall health and fitness in firefighters, but evidence beyond clinical and performance outcomes is needed before fire departments invest in and successfully adopt health promotion programs.

**Objective:**

This mixed methods pilot study sought to pair clinical and performance outcomes with participants’ qualitative feedback (eg, participants’ enjoyment, lifestyle behavior changes, and team structure) with the goal of informing recommendations for future programs.

**Methods:**

Professional firefighters participated in a 14-week occupational exercise training program with assessments conducted pre- and posttraining. Clinical outcomes included weight, BMI, body fat percentage, resting heart rate, systolic blood pressure, and diastolic blood pressure. Performance outcomes included the sharpened Romberg balance test, 1-repetition maximum leg press and bench press, graded exercise test (estimated VO_2max_), knee range of motion, shoulder flexibility, and hamstring flexibility. Self-administered surveys (Short Form-36, International Physical Activity Questionnaire, Barriers Self-Efficacy Scale, and Barriers to Being Active Quiz) were completed. In 3 private focus groups of 3 to 4 participants, firefighters' experiences in the training program and their health behaviors were explored.

**Results:**

Male firefighters (n=14; age: mean 36.4, SD 2.6 years) completed 20 training sessions. There were no significant changes to weight (*P*=.20), BMI (*P*=.15), body fat percentage (*P*=.16), systolic blood pressure (*P*=.12), estimated VO_2max_ (*P*=.34), balance (*P*=.24), knee range of motion (left: *P*=.35; right: *P*=.31), or hamstring flexibility (*P*=.14). There was a significant decrease in diastolic blood pressure (*P*=.04) and significant increases in shoulder flexibility (*P*<.001) and leg press 1-repetition maximum volume (*P*=.04). Participants reported improvements in overall health, endurance, flexibility, and mood as well as improvements to team environment and health behaviors around the station; however, there was a decline in overcoming barriers to physical activity.

**Conclusions:**

A 14-week program of exercise training in firefighters elicited improvements in clinical, performance, and self-reported physical activity outcomes. This occupational exercise training program for firefighters increased time spent exercising, improved team building, and led to physical and mental health benefits. Results from this pilot study set a broad, informed, and meaningful foundation for future efforts to increase firefighter participation in occupational fitness programs.

## Introduction

Heart disease is the number one cause of firefighter fatalities [1] accounting for nearly 50% of all firefighting-related deaths [2,3]. In general, firefighters have high rates of cardiovascular disease risk factors such as hypertension and obesity [4] contributing to their high overall risk of death from heart disease [5]. Not only would an acute event harm the individual, but any health condition that limits a firefighters’ performance compromises the safety of colleagues and the community. Poor physical fitness is associated with a higher risk of cardiovascular disease and acute myocardial events [6]. Carrying heavy gear and wearing restrictive clothing in extreme heat while performing the high intensity work of firefighting elicits significant physiological stress which, when paired with poor fitness, can lead to line of duty cardiovascular disease events [7]. Furthermore, poor fitness is associated with socioeconomic consequences due to the benefits awarded to responders who die or are disabled by cardiovascular disease or who are affected by work-related orthopedic problems [8]. Therefore, improving and maintaining overall health and physical fitness is critical to the safety and longevity of firefighters and those they serve.

Any amount of exercise is beneficial in reducing cardiovascular disease risk, reducing all-cause mortality up to 40% [9,10], and emergency personnel who engage in higher levels of physical activity are more physically fit than less active peers [11]. Regular exercise training elicits adaptations that can improve job performance, reduce the risk of cardiovascular events, and prevent musculoskeletal injuries [12-17]. Furthermore, exercise is beneficial for firefighters, significantly improving body fat percentage, aerobic capacity, endurance, strength, and power [18]. High fitness levels are correlated with improved job performance during real and simulated firefighting activities [19-21]. Unfortunately, infrequent physical activity is common in fire service, and most departments do not mandate regular exercise or require that firefighters maintain fitness standards [22].

One means of improving overall health and fitness in firefighters is through the implementation of departmental or occupational fitness programs [22,23]. Indeed, firefighters within departments that incorporate a wellness or fitness program are less likely to be obese, more likely to meet endurance standards for firefighting, more likely to have higher cardiorespiratory fitness, more likely to express higher job satisfaction, and are less likely to smoke or be diagnosed with an anxiety disorder [24]. While the National Fire Protection Association Needs Assessment emphasizes the importance of health and wellness programs, only 30% of departments have such a program [25]. Several investigators have issued a call to action for health and fitness professionals to assist fire departments in implementing programs for firefighters that improve fitness and reduce cardiovascular disease risk factors [26,27].

For firefighter departments to adopt and invest in occupational wellness programs that promote exercise training, evidence of success beyond the exhaustively reported [9-24] clinical and performance outcomes is needed. To date, the comprehensive influence of these programs has not been fully explored including the role of these programs in inciting permanent behavior change or their influence on team structure or building. Indeed, others have noted that exercise patterns are influenced by the culture of fire stations [28], but this has not been explored.

Finally, there still exists a gap in understanding how best to intervene in firefighters’ health promotion [28-30], and participant feedback has not been reported within the literature. We evaluate the influence of an occupational fitness program on qualitative outcomes including participants’ enjoyment, lifestyle behavior changes, and team structure. To our knowledge, this is the first study to incorporate mixed method analyses of clinical and performance outcomes and firefighters’ experiences and feedback.

## Methods

### Design

This semiexperimental, community-based participatory intervention research pilot study was approved by the institutional review board of West Virginia University (1812386641). Briefly, a local firefighter captain, concerned about the apparent poor physical fitness of the firefighters under his command, approached faculty in the division of Exercise Physiology about implementing an exercise training program within his 3 companies. The investigators (exercise physiology faculty members at West Virginia University), assisted by trained undergraduate exercise physiology student interns, developed and implemented an occupational exercise training program for the firefighters. Clinical, performance, and survey results were collected before and after a 14-week exercise training intervention. At the end of the intervention, the firefighters participated in focus groups to capture impressions of the intervention. While the captain strongly encouraged all firefighters under his directive to participate, enrollment into the research project was voluntary. Written and verbal consent were obtained from all participants.

### Intervention

The exercise training program ran from mid-January through mid-April 2019. Due to the firefighters’ irregular schedules, training occurred at least once, but usually twice weekly either at the nearest fire station (equipped with a small gym facility) or in the Human Performance Laboratory (a fitness and research facility in the division of exercise physiology) at West Virginia University. Four student interns trained 3 to 4 firefighters each. The student interns developed and prescribed exercises targeted to improving functional outcomes in firefighters (eg, getting in and out of the truck, carrying heavy loads up and down stairs, etc) and which incorporated strength, muscular endurance, cardiovascular, flexibility, and agility exercises.

Participants visited the laboratory for assessments before starting and after completing the 14-week training program. Participants were instructed to arrive in athletic shoes and comfortable clothing as well as to abstain from vigorous exercise for 24 hours and caffeine and tobacco for 4 hours prior to testing. All assessments were conducted pre- and postintervention with the exception of the health history questionnaire, which was completed after consent but before physical assessments.

### Assessment Measures

#### Overview

Before arriving to the laboratory, all participants completed a health history questionnaire to assess information related to disease risk, medication use, and lifestyle behaviors (eg, physical activity, nutrition, stress). Upon arrival, to the laboratory, a resting 12-lead electrocardiogram (ECG) was performed by a trained technician and reviewed by a clinical exercise physiologist for evidence of irregular myocardial contractility or arrhythmia. An experienced technician measured resting seated brachial blood pressure of the right arm using a stethoscope and sphygmomanometer and resting heart rate by palpation at the radial artery. The clinical exercise physiologist reviewed the health history questionnaire and assessment results and followed American College of Sports Medicine safety guidelines to determine whether it was safe for participants to exercise [31]. Any evidence of risk required that the participant follow up with their physician before continuing with preassessments or starting the exercise program.

#### Anthropometrics

Standing height (±0.1 cm) was measured with a stadiometer, and body weight (±0.1 kg) was measured with a calibrated body mass scale with the participant barefoot and in light clothing. Body mass index (BMI) was calculated from height and weight (kg/m^2^). Percentage body fat was measured with air plethysmography using a BodPod (Cosmed USA Inc).

#### Balance Test

The sharpened Romberg balance test was administered to participants by a trained technician. Participants stood with feet heel-to-toe, arms crossed over their chest, with eyes open for 60 seconds or until the participant moved one or both feet. If the participant successfully completed 60 seconds without moving, the test was repeated with eyes closed.

#### Strength Measures

Participants warmed up by walking on a treadmill at a self-selected speed before performing lower and upper body maximum strength assessments. Lower body strength was assessed with 1-repetition maximum leg press, and upper body strength was assessed with 1-repetition maximum bench press. For the bench press machine (Body Masters Sports Industries Inc), the handle was lowered to chest level. A repetition began with the handle at the level of the chest with no resistance and was completed when the elbows reached full extension. For the leg press (Body Masters Sports Industries Inc) machine, the participant’s feet were placed on the platform shoulder width apart and with the toes slightly rotated outwards. The platform was then adjusted to produce a 90-degree bend at the knee joints. Repetitions began with the knee flexed with no resistance and ended with the knee nearly completely extended. The 1-repetition maximum was recorded as the heaviest weight lifted only once. In the event that the participant could lift the entire weight stack more than once, they were instructed to perform repetitions until voluntary fatigue and maximal 1-repetition maximum volume was calculated as weight unit × repetitions.

#### Submaximal Graded Exercise Test

A Cornell graded submaximal treadmill test was administered by trained technicians and supervised by a clinical exercise physiologist. Participants completed the staged protocol until 85% of heart rate reserve or volitional fatigue. A clinical exercise physiologist monitored a 3-lead ECG for evidence of myocardial incontractility or irregular electrical activity during the test. Heart rate, blood pressure, and rate of perceived exertion were measured at each stage to monitor participant safety (data not reported). VO_2max_ was estimated using established equations [31].

#### Flexibility and Range of Motion

Measurements of maximal knee extension and flexion with a goniometer enabled calculation of knee range of motion (degree of maximal extension–degree of maximal flexion). Knee range of motion was measured with the participant lying supine and the ipsilateral hip and knee extended throughout the examination. The axis of the goniometer was centered on the lateral femoral condyle. The stationary arm was aligned along the femoral shaft toward the greater trochanter, while the moving arm was aligned with the fibula downward toward the lateral malleolus. Knee flexion was measured first by flexing the hip to 90°, and then instructing the participant to draw the heel as close to the buttocks as possible. From this position, the participant actively extended the knee until resistance was felt to measure knee extension.

For shoulder flexibility, participants lay in a prone position on the floor, forehead on the ground, and arms extended holding an 18-inch (45-cm) stick with both hands shoulder width apart. The participants raised the stick as high as possible off the floor while keeping the forehead on the ground and a technician measured and recorded the vertical distance achieved from the best of 3 trials.

A sit-and-reach test was performed to assess hamstring flexibility. Briefly, participants sat on the floor with knees extended and feet against the sit-and-reach box and at right angles to the floor. The participants reached forward as far as possible with knees locked and hands one on top of the other. The maximal reach (distance between the box's zero point and participants' reach point) from the best of 3 trials was recorded.

#### Surveys

Self-administered surveys—the Short Form-36 [32], International Physical Activity Questionnaire (IPAQ) [33], Barriers Self-Efficacy Scale (BARSE) [34], and Barriers to Being Active Quiz (BBAQ) [35]—were completed by participants in private.

The Short Form-36 is a multipurpose, short form, validated health survey of 36 questions. The Short Form-36 has 8 scaled scores; the scores are weighted sums of the questions in each section. Scores range from 0 to 100 with lower scores indicating more disability and higher scores indicating less disability. Sections include vitality, physical functioning, bodily pain, general health perceptions, physical role functioning, emotional role functioning, social role functioning, and mental health.

The IPAQ covers 4 domains of physical activity: work-related, transportation, housework/gardening, and leisure time activity including questions about time spent sitting as an indicator of sedentary behavior. In each domain, the number of days per week and time per day spent in both moderate and vigorous activity are recorded. At work, during transportation and in leisure time, walking time is also included. Moderate intensity was defined as 4 MET (metabolic equivalent task) and vigorous intensity was defined as 8 MET. Outcome measures used were MET hours per week

The Barriers Specific Self-Efficacy Scale is a 13-item questionnaire with 11-point Likert scale response options designed to assess participants’ perceived capabilities to exercise. For each item, participants indicate their confidence about executing their exercise behavior. Possible scores range from a minimum self-efficacy score of 0 to a maximum possible score of 100.

The Barriers to Being Physically Active Quiz is a 21-item measure assessing barriers to physical activity including lack of time, social influence, lack of energy, lack of willpower, fear of injury, lack of skill, and lack of resources. Each domain contains 3 items, with a total score range of 0 to 63. Respondents rate the degree of activity interference on a 4-point scale, ranging from 0 (very unlikely) to 3 (very likely).

#### Qualitative Methods

Qualitative research methods, including focus groups, can provide rich, descriptive data that can be missed when using quantitative methods. Furthermore, qualitative approaches can enhance collaboration with working people by offering them a forum for discussing the particular problems of their employment and health and for developing feasible intervention programs [36-38]. A semistructured protocol following standard focus group guidelines [39] was conducted by trained researchers [39,40]. Three private focus groups of 3 to 4 participants responded to a series of 9 questions, some with probing follow-ups, which explored their experiences in the training program and their health behaviors. A trained note taker took comprehensive notes on a laptop computer at each focus group, and proceedings were digitally audiorecorded. These notes were reviewed by the focus group moderator for clarity, thoroughness, and accuracy.

### Analysis

For quantitative data, descriptive statistics summarizing clinical, performance, and numerical questionnaire data were analyzed using Excel (Microsoft Inc). Differences between pre- and postassessments were assessed using independent one-tailed *t* tests with significance set at *P*<.05.

Grounded theory allows for an inductive theory–building approach, one that does not require a prior theory [41]. Using a heuristic or common-sense approach based in grounded theory, 2 independent researchers (with a tiebreaker when necessary) analyzed the content of focus group discussions to identify common themes. The researchers discussed independent content analysis findings; however, due to the limited sample size, saturation of repetitive concepts (ie, point at which no new information, trends, or themes emerge from data) was not achieved [42].

## Results

### Clinical and Performance Results

Of 16 men who consented, one participant was excluded from participating because of evidence of myocardial dysfunction. A total of 15 men started the training program, but one withdrew due to an orthopedic injury incurred outside the research study. Therefore, 14 Caucasian males (mean 36.4, SD 2.6 years old) completed 20 training sessions over a total of 14 weeks; 2 participants were taking blood pressure medications, and 6 had a history of orthopedic injuries that did not prohibit their participation. Clinical outcomes are presented in Table 1. There were no significant changes to weight (*P*=.20), BMI (*P*=.15), percentage body fat (*P*=.16), or systolic blood pressure (*P*=.12). There was a significant decrease in diastolic blood pressure (*P*=.04), and the reduction in resting heart rate was trending toward significance (*P*=.08).

**Table 1:** Clinical outcomes.

Outcome	Pre, mean (SD)	Post, mean (SD)	*P* values
Height (cm)	179.8 (1.7)	N/A^a^	N/A
Weight (kg)	96.5 (4.9)	99.0 ( 4.5)	.20
BMI (kg/m^2^)	30.1 (1.8)	31.0 (1.5)	.15
Body fat (%)	31.7 (2.6)	30.9 (2.3)	.16
Systolic blood pressure (mmHg)	129 (2)	125 (3)	.12
Diastolic blood pressure (mmHg)	93 (6)	78 (2)	.04
Resting heart rate (bpm)	80 (2)	76 (3)	.08

^a^N/A: not applicable.

From the health history questionnaire, average responses to physical activity patterns indicated that participants were mostly sitting or standing at work and that they perceived their levels of physical fitness to be below average. Half of the participants exercised 3-5 days/week for at least 6 months, but half reported no regular exercise. Participants reported the fat content in their diet was slightly above average, 10 out of 14 (71%) participants regularly consumed alcoholic beverages, and 11 out of 14 (79%) regularly consumed caffeine. Most (13/14, 93%) reported average to above average job stress.

Physical performance outcomes are reported in Table 2. All participants completed the sharpened Romberg balance test with eyes open. There were no significant changes in estimated VO_2max_ (*P*=.34), knee range of motion (left: *P*=.35; right: *P*=.31), or hamstring flexibility (*P*=.14). Shoulder flexibility (*P*<.001) and leg press 1-repetition maximum volume significantly increased (*P*=.04), and the increase in bench press 1-repetition maximum volume was trending toward significance (*P*=.07).

**Table 2:** Physical performance outcomes.

Outcome	Pre, mean (SD)	Post, mean (SD)	*P* values
Romberg balance closed (seconds)	58.9 (1.1)	57.0 (2.3)	.24
Estimated VO_2max_ (mL/kg/minute)	55.8 (3.3)	57.3 (4.0)	.34
Left leg knee range of motion (degrees)	142.1 (2.3)	141.1 (3.6)	.35
Right leg knee range of motion (degrees)	142.4 (2.9)	140.5 (3.5)	.31
Shoulder flexibility	14.6 (1.1)	18.8 (1.2)	<.001
Hamstring flexibility	12.5 (1.0)	13.3 (0.9)	.14
Leg press 1-repetition maximum volume (repetitions × weight unit)	5896 (1119)	7710 (1315)	.04
Bench press 1-repetition maximum volume (repetitions × weight unit)	2001 (480)	2781 (678)	.07

### Survey Data

Changes in IPAQ responses indicated more time spent doing vigorous physical activity as part of work (METminutes/week—pre: mean 1971, SD 514; post: mean 5920, SD 2105; *P*=.03), more time spent walking as part of work (METminutes/week—pre: mean 3117, SD 722; post: mean 5074, SD 934; *P*=.03), and less time spent sitting on weekdays (minutes—pre: mean 287, SD 28; post: mean 214, SD 32; *P*=.04).

Overall, there were no differences in group averages for the Short Form-36 (*P*=.50), BBAQ (*P*=.44), or BARSE (*P*=.19). However, changes in Short Form-36 showed significantly improved responses to “In general, would you say your health is” (*P*=.048), and the responders felt their health had improved from “about the same” to “somewhat better now” when asked to compare their health to a year ago (*P*=.007). There was a significant improvement in average responses to “How much of the time during the past 4 weeks have you felt so down in the dumps that nothing could cheer you up?” (*P*=.04). However, changes in the BARSE indicated a significant decline in overcoming barriers to physical activity in the domains of lack of time (*P*=.03) and lack of energy (*P*=.045). In the BBAQ, there were significant declines in response to “I believe that I could exercise 3 times per week for the next 3 months if I had to exercise alone” (*P*=.04) and “I believe that I could exercise 3 times per week for the next 3 months if I was under personal stress of some kind” (*P*=.048).

### Qualitative Results

Focus group themes are presented in Table 3. The firefighters reported positive feelings prior to starting the training program and liked the idea, citing enthusiasm of gaining fitness:

I wanted to regain some of what I’ve lost over the years.

Many expressed reservations; a few attributed their reservations to disinterest, but most attributed it to nervousness, specifically not knowing what to expect and feeling out of shape.

By the end of the training program, the impressions were positive, and many expressed interest in continuing the program:

I really hope they can keep a program and designated times for that.

It was even recommended that the program be extended throughout the entire firefighting department:

I would like to see the department mandate a time in the shift for PT [physical training]… It would push a lot of guys into physical training.

Firefighters liked having the interns as trainers and appreciated the interns’ preparation but suggested assigning intern trainers specific to participants’ interests (cardiovascular versus strength). They liked the variety of exercises (eg, balance exercises and circuit training) and learning new exercises (eg, planks), but many suggested including more task-specific exercises and training:

Flipping the tire was more incorporative of what we actually do.

Include a routine that would have a fireman drill.

While the firefighters enjoyed training in the fitness facility for “getting out of the station” most felt, compared with training at the station, that it posed difficulties logistically. Other recommendations were to have more training rather than once or twice a week:

I feel like I would have gotten more out of it.

Other than merely receiving exercise programming from the intern trainers, the firefighters reported receiving positive motivation but with different styles of support (eg, cheerleading versus drill sergeant). They felt being divided into groups inspired a bit of competition which helped with team motivation.

In terms of exercising outside of meeting times, some of the interns had prescribed “homework” to their group to encourage the firefighters to exercise on off days; the firefighters that were prescribed outside exercise reported completing it. Many of the younger firefighters reported incorporating extra exercises outside of training times of their own volition, but the older firefighters cited lack of time and motivation as barriers:

Life seemed too busy to be able to do it...

...easier to work out in a group...

Some thought they were more likely to incorporate exercise in the future using what they learned from the intern trainers.

Physical performance of their occupation was improved by increased endurance,

I don’t get as winded when I’m walking or doing things...

and many firefighters noted climbing stairs with their gear was significantly easier. Several reported less mental fatigue citing general tasks felt easier:

Any time I put my gear on you can tell a difference.

Other reported benefits were increased flexibility, better recovery and less soreness from firefighter runs or calls, and “more confidence in skills.”

The firefighters reported improvements around the station including more support and improved attitudes toward fitness. Some firefighters reported improvements in health behaviors among the group, including a noticeable influence on nutrition:

It’s definitely improved with eating better and feeling better.

More fruit instead of sugary foods...

...less soda, more water...

There was more talk around the station about exercise, and many reported improved team building,

...play together, stay together...

...seeing guys from other companies helped change the little bickering between them...

This sentiment was reported by the captain and echoed by the group. Many firefighters noted more positive attitudes in their colleagues, which was also reported outside of work; many had better moods and attitudes:

Normally you leave here cranky, irritated and now I leave here in a good mood.

Outside of work, firefighters reported several wellness benefits including feeling better overall, being more productive, and being more inclined to exercise and eat healthier when away from the station.

Overall, there was an increased affinity for exercise with plans to continue into the summer. All firefighters would recommend this program to other companies because it increased overall exercise time, incorporated accountability and team building, and led to physical and mental health benefits:

It was hard, but you get paid to have a personal trainer.

**Table 3:** Focus groups themes.

Topics and themes		Findings
**When reflecting to the start of the program:**		
	Positive feelings:	Looked forward to gaining fitness
	Some reservations:	Uncertainty, nervousness, and some disinterest
**Training program feedback:**		
	General feeedback:	Liked having interns as trainers and their positive motivation Enjoyed the variety of exercises, learning new exercises, and competition between groups Interested in continuing
**Self-reported outcomes:**		
	Performance:	Increased endurance Less mental fatigue Increased flexibility Better recovery More confidence
	Around the station:	Increased support from peers More positive attitudes Improved attitudes toward fitness Improved health behaviors Increased team building,
	At home and in their personal lives:	Better mood Feeling better overall Feeling more productive More inclined to exercise and eat healthier when away from the station
**Moving forward:**		
	Why they recommend participation:	Increased overall exercise time Accountability Team building Physical and mental health benefits
	Specific recommendations:	Implement throughout the department Incorporate task-specific exercises and training Train specific to participants interests (cardio vs. strength) Incorporate more days of training

## Discussion

While physiological (performance and clinical) outcomes have been widely reported [9-24], the comprehensive influence of occupational exercise training programs has not been fully explored, and participant feedback has not been reported within the literature. This semiexperimental, community-based participatory intervention pilot study found reported improvements in overall health, endurance, flexibility, and mood, as well as improvements to team environment and health behaviors around the station; however, there was a decline in overcoming barriers to physical activity. Positive reports as well as future recommendations indicate the success of this pilot study and promising avenues for implementing similar and more wide-reaching programs moving forward.

Combined interventions have been shown to be most effective in improving cardiovascular health in emergency personnel [43]. Therefore, in this pilot study, professional firefighters received personalized exercise recommendations, which incorporated strength, muscular endurance, cardiovascular, flexibility, and agility exercises, designed to improve functional outcomes. There was a significant reduction in diastolic blood pressure (*P*=.04), while resting heart rate reductions were trending toward significant (*P*=.08), which is in agreement with previous findings showing a 4-week circuit training program can improve vascular outcomes in firefighters [44]. Indeed, many fitness variables, including resting heart rate, contribute to firefighters performance in simulated firefighting tasks [45] suggesting the clinical outcomes in this study could translate to improved occupational performance and overall reductions in cardiovascular disease risk. This is supported by survey and focus group responses which indicated improvements in self-perceptions of overall health. Importantly, firefighters must excel in multiple areas of physical fitness including flexibility to climb in and out of the truck, power to perform forced entry maneuvers, muscular and cardiovascular endurance to carry equipment up flights of stairs, and strength to advance hose lines [27]. For performance outcomes, there was a significant increase in shoulder flexibility (*P*<.001) and leg press 1-repetition maximum (*P*=.04), and bench press 1-repetition maximum was trending toward significant (*P*=.07). Firefighting tasks are highly associated with muscle strength and endurance [45-50], and though firefighting tasks were not directly assessed, the qualitative responses from focus groups indicated improved endurance and flexibility.

Many clinical and performance measures were unchanged after the intervention, which could be attributed to the small sample size or insufficient exercise stimulus. For example, estimated VO_2max_ was unchanged, perhaps because the exercise stimulus from meeting once or twice a week was insufficient to elicit the necessary physiological aerobic adaptations to improve maximal oxygen consumption. Additionally, outcomes such as knee range of motion and balance were within normal ranges at baseline and would have been unchanged even with a rigorous exercise training program.

Independent of physiological outcomes, participants reported improvements in performance and overall health, including increased vigorous activity and more walking at work as well as reduced sitting time during weekdays. In terms of exercising outside of scheduled training times, the firefighters who were prescribed outside exercise reported completing it, and many of the younger firefighters reported incorporating extra exercise outside of training times of their own volition. These findings were supported by previous qualitative research that found younger firefighters were more health conscious [36]. Furthermore, the firefighters reported improvements around the station, including more support and improved attitudes toward fitness, which is supported by previous research that found participation in fitness training improves the perceptions of exercise in emergency personnel [51].

In addition to fitness, the firefighters reported improvements in other health behaviors, such as nutrition. Despite the lack of direct measures (eg, pedometers, nutrition intake surveys, etc), these findings are evidence of improvements to lifestyle behaviors suggesting the goal of inciting permanent behavior change was supported. However, survey data indicated a significant decline in overcoming barriers to physical activity in the domains of lack of time (*P*=.34) and lack of energy (*P*=.045) and in response to “I believe that I could exercise 3 times per week for the next 3 months if...” There were significant declines in response to “I had to exercise alone” (*P*=.042) and “I was under personal stress of some kind” (*P*=.048). Despite favorable impressions of exercise, some of these barriers (eg, lack of time and motivation) were echoed in the focus groups. Therefore, the goal of inciting permanent behavior change cannot conclusively be supported.

Participants reported improvements in mood and attitude as assessed by surveys and focus groups. Interestingly, in the focus groups, respondents cited improvements in their own moods and attitudes at work and home as well as improvements in those of their colleagues. Since firefighting has been shown to be associated with considerable psychological stress [52], the improvements in mood could indicate improvements in overall stress. While not a primary objective of this pilot study, these findings are certainly relevant to recommendations for future programs.

In focus groups, many participants reported improvements to the team environment: the captain felt that “seeing guys from other companies helped change the little bickering between them” and this sentiment was echoed by the group. One unforeseen mechanism driving this influence was the random allocation of firefighters to training groups: training with firefighters outside their unit promoted team building across the company. Additionally, the training sessions inspired a bit of competition among firefighters which helped with team motivation as well. Others [36] have noted that the culture of the fire station can influence exercise patterns and that firefighters work in close-knit teams (eg, sharing meals and training together) suggesting social support may be an important factor to behavior change. While previous research [43] has cited the potential of occupational health promotion programs to take advantage of the existing team structure within fire departments, to our knowledge, this is the first pilot study to directly assess this outcome. Working as a team is critical to successful firefighting and ensures the safety of the responders and those they serve. Therefore, the potential for an occupational exercise training program to improve team structure has significant implications for program implementation moving forward.

Impressions of this pilot program were positive and many expressed interest in continuing the program, even requesting more days per week of training. The design of the program, including having the interns as trainers and the variety of exercises, were received favorably. A primary outcome of this study was to inform recommendations for future programmatic implementation of occupational firefighter fitness programs. All firefighters would recommend this program to other companies because it increased overall exercise time, incorporated accountability and team building, and led to physical and mental health benefits. The participants recommended that the program be extended throughout the entire firefighting department, but many suggested including more task-specific exercises and training as well as limiting training to the fire stations rather than outside training facilities.

While the findings of this pilot study suggest support for the feasibility of this occupational exercise training program, the study is not without limitations. This project was implemented in a relatively urban setting within Appalachia, and because the individualized exercise programs were implemented by students as part of their coursework, this personal training was free to the firefighters. Also, because the fire captain specifically requested this occupational exercise program, it was a community-based participatory research study. Therefore, the findings of this study may not be generalizable to other settings such as rural areas with limited resources, minority populations, or fire companies that lack leadership commitment to the program. Additionally, a significant limitation of this pilot study was the small sample size, which can be associated with low reproducibility [53]. However, as previously described, the clinical and performance outcomes of this study are not novel and have been exhaustively reported in the literature [9-24]. Instead, these quantitative outcomes were included, in part, to demonstrate that the exercise intervention, though limited, with only 20 session over 14 weeks, was sufficient to elicit physiological changes (eg, improvements in strength and blood pressure). Additionally, the intervention is reflective of the realistic time commitment that can be expected from fire department occupational fitness programs: most departments have schedules in which firefighters are on duty 2 times per week, thus any training program implemented by the department during shift work would be limited in frequency of training sessions per week. While this may not allow for a more traditional training protocol (occurring 3 times per week), in this study, because sessions lasted 90 minutes, the training program met common recommendations of 150 minutes of exercise per week [54].

The novelty of this pilot program was the qualitative participant feedback including participants’ enjoyment, lifestyle behavior changes, and team structure with the goal of informing recommendations for future programs. We found improvements in overall health, endurance, flexibility, and mood, as well as improvements to team environment and the health behaviors around the station suggesting that the implementation of this fitness program was well received by participants.

Moving forward, this project will be expanded throughout the city’s fire department, offering free personal training from university exercise physiology students to all firefighters. While this study found favorable outcomes among participants, it is a feasibility study, and future research is warranted. In particular, qualitative feedback from fire department leadership that captures barriers to department-wide exercise training programs is needed before permanent programs can be implemented. Additionally, this study trained professional firefighters, but similar work could be evaluated in volunteer firefighters. The outcomes presented here are used to attempt to understand how best to intervene in firefighters’ health promotion [28-30], but the role of exercise training in permanent behavior change and the influence of group participation on team structure and building should be more thoroughly explored. In this study, firefighters received personalized exercise guidance designed to improve functional outcomes by incorporating strength, muscular endurance, cardiovascular, flexibility, and agility exercises into the exercise programs; however, future research could evaluate a firefighting task-specific exercise training program with the goal of improving task-specific performance and overall effect on job performance.

The 14-week exercise training program improved clinical (diastolic blood pressure and resting heart rate) and performance (1-repetition maximum leg and bench press) outcomes, as well as self-reported physical activity (more vigorous activity and walking, less sitting time). Improvements in overall health, endurance, flexibility, and mood as well as improvements to team environment and the health behaviors around the station were reported; however, a decline in overcoming barriers to physical activity was also found. Overall, the impressions of this pilot program were positive because it increased time spent exercising, improved team building, and led to physical and mental health benefits. Recommendations moving forward are to extend the program throughout the fire department and to include more task-specific exercises and training.

## Abbreviations

BARSE: Barriers Self-Efficacy Scale
BBAQ: Barriers to Being Active Quiz
ECG: electrocardiogram
IPAQ: International Physical Activity Questionnaire
MET: metabolic equivalent task
